# Measuring Ambiguity in HLA Typing Methods

**DOI:** 10.1371/journal.pone.0043585

**Published:** 2012-08-29

**Authors:** Vanja Paunić, Loren Gragert, Abeer Madbouly, John Freeman, Martin Maiers

**Affiliations:** 1 Bioinformatics Research, National Marrow Donor Program, Minneapolis, Minnesota, United States of America; 2 Department of Computer Science and Engineering, University of Minnesota, Minneapolis, Minnesota, United States of America; Centro Cardiologico Monzino IRCCS, Italy

## Abstract

In hematopoietic stem cell transplantation, donor selection is based primarily on matching donor and patient HLA genes. These genes are highly polymorphic and their typing can result in exact allele assignment at each gene (the resolution at which patients and donors are matched), but it can also result in a set of ambiguous assignments, depending on the typing methodology used. To facilitate rapid identification of matched donors, registries employ statistical algorithms to infer HLA alleles from ambiguous genotypes. Linkage disequilibrium information encapsulated in haplotype frequencies is used to facilitate prediction of the most likely haplotype assignment. An HLA typing with less ambiguity produces fewer high-probability haplotypes and a more reliable prediction. We estimated ambiguity for several HLA typing methods across four continental populations using an information theory-based measure, Shannon's entropy. We used allele and haplotype frequencies to calculate entropy for different sets of 1,000 subjects with simulated HLA typing. Using allele frequencies we calculated an average entropy in Caucasians of 1.65 for serology, 1.06 for allele family level, 0.49 for a 2002-era SSO kit, and 0.076 for single-pass SBT. When using haplotype frequencies in entropy calculations, we found average entropies of 0.72 for serology, 0.73 for allele family level, 0.05 for SSO, and 0.002 for single-pass SBT. Application of haplotype frequencies further reduces HLA typing ambiguity. We also estimated expected confirmatory typing mismatch rates for simulated subjects. In a hypothetical registry with all donors typed using the same method, the entropy values based on haplotype frequencies correspond to confirmatory typing mismatch rates of 1.31% for SSO versus only 0.08% for SBT. Intermediate-resolution single-pass SBT contains the least ambiguity of the methods we evaluated and therefore the most certainty in allele prediction. The presented measure objectively evaluates HLA typing methods and can help define acceptable HLA typing for donor recruitment.

## Introduction

The Human Leukocyte Antigen (HLA) gene system on Chromosome 6 is one of the most polymorphic regions of the human genome and one of the most extensively studied regions due to its importance in transplantation and association with autoimmune, infectious and inflammatory diseases [1]–[3]. The HLA region contains genes that encode protein products crucial for adaptive immune response, and its high genetic polymorphism allows the immune system to fight a variety of pathogens. The HLA gene system plays a crucial role in hematopoietic stem cell transplantation (HSCT), where patients and donors are matched with respect to their HLA genes, in order to maximize the chances of successful transplant [4]. Developments in DNA based typing methods have seen a large increase in new HLA alleles being identified each year, at an average rate of more than one new allele discovered per day [5]–[7]. Discovered alleles and their sequences along with the most recent information on HLA region are catalogued in the IMGT/HLA database [8]. As of the first quarter of 2012, more than 1700, 2300, and 1000 alleles have been discovered for the class-I HLA-A, -B, and class II HLA-DRB1 loci, respectively (http://www.ebi.ac.uk/imgt/hla/) [9].

The high polymorphism in HLA presents a challenge when it comes to typing HLA genes. The typing has historically been performed using serological antibody tests, which are able to identify HLA protein variants on the surface of the cell using antigen-specific antibodies [10]. Serology has been widely replaced with DNA-based typing methods due to its inability to identify all specific products of the HLA alleles. It is shown that in order to improve the clinical outcome of HSCT from an unrelated donor, it is essential to identify and match patient and donor's HLA genes at the allele level [11]–[13].

DNA-based methods identify HLA alleles by interrogating the nuclear DNA sequence and can result in different levels of ambiguity depending on typing methodology or test kits used. HLA typing methods, their corresponding formats and abbreviations used in this paper are given in Table 1. Some of the widely used molecular methods for typing HLA genes, as defined in American Society for Histocompatibility and Immunogenetics Standards (ASHI), are a nucleic acid-based typing method using sequence-specific oligonucleotide hybridization (SSO) [14], [15], a nucleic acid amplification-based typing method using sequence-specific priming (SSP) and sequence-based typing (SBT) [16]. Even though DNA-based technology improved identification of specific alleles, HLA typing results reported by testing laboratories are still commonly resolved to a certain level of ambiguity, rather than to an exact allele assignment [5]. Exact high resolution HLA typing can be costly and laborious with the large and rapidly growing number of described HLA alleles, which sometimes cannot be easily distinguished with existing high-throughput typing methods. Ambiguous allele assignments are produced either due to failure to interrogate all polymorphic positions, or due to a lack of phase between polymorphisms within a locus because of diploid sequence reads (or both).

**Table 1:** HLA typing formats reported by NMDP contract typing laboratories.

Typing Method	Description			Abbreviation	Example Typing
**Serology**	Identifies HLA protein on the cell surface using antigen-specific antisera.	Broad antigen typing		SERO	A9, A28
		Split antigen typing			A24, A68
**DNA**	Identifies HLA alleles by interrogating DNA	Allele family level	Two digit resolution	DNA2	A*24:XX, A*68:XX
		Sequence specific oligonucleotides	Allele codes	SSO	A*24:AER, A*68:GM AER = 02/03/04/05 GM = 01/02/03/04/05
		Sequence based typing	Single pass SBT	SBT	A*24:02,A*68:01 or A*24:03,A*68:01 or A*24:04,A*68:01 or A*24:05,A*68:01
		*High resolution [45]	Exact alleles	High resolution	A*24:02, A*68:01

* Since the expression has not been confirmed for the majority of alleles described in IMGT/HLA, we use high-resolution here to denote the amino acid sequence of the exons encoding the antigen binding domains.

In HSCT the selection of donors for a patient in need of a transplant is based primarily on HLA matching, and the lower the ambiguity of typing the easier it is to determine the probability of allele level match during the donor search [17]. In order to facilitate rapid identification of matched donors for HSCT several methods have been proposed to infer unknown phase and allele assignment [4], [18]. They typically employ statistical methods and the unique properties of the HLA region, such as its high linkage disequilibrium, in order to estimate haplotype frequencies and predict the most likely haplotype assignment for an individual with an HLA typing consisting of a set of ambiguous allele pairs. HLA typing with less ambiguity on average gives fewer high-probability phased high resolution haplotypes. We aim to measure per-locus ambiguity resulting from several HLA typing formats across four continental populations using an information theory-based measure, Shannon's entropy [19].

Some previous work has been done in measuring typing ambiguity – first a measure developed by Helmberg et al. [20] and more recently, the first application of Shannon's entropy to this problem by Cano [21]. Helmberg proposed a characterization of HLA typing kits using a frequency inferred typing (FIT) index. A FIT index describes the probability of correct allele pair assignment for an ambiguous typing result, and is calculated as the negative log of the probability of a wrong allele pair prediction. This probability is equal to the sum of products of all allele pairs that share the same typing pattern as the selected pair. The limitation of the FIT index is that it does not take into account the distribution of allele frequencies beyond the most likely assignment. We provide more detail on the FIT index in Supporting Information (Text S1). The concept of measuring ambiguity in HLA typing using entropy was first presented by Cano in [21]. They used population-specific allele frequencies and several SSO typing examples to demonstrate the utility of the measure.

Both previous typing ambiguity studies used allele frequencies in their computations and, as we will later show, fail to demonstrate the advantage of linkage disequilibrium information contained in haplotype frequencies when it comes to reducing ambiguity and improving predictions of patient-donor matching. We use haplotype frequencies and show that the ambiguity is reduced considerably compared to using allele frequencies, proving that this advance in strategy for identifying matched donors has had a significant positive impact. In addition, we take this methodology further and use it to evaluate several different typing methods, and directly compare them with respect to the inherent ambiguity measured by entropy. To measure the impact of the typing method ambiguity in more relatable terms, we also developed a measure we call Confirmatory Typing (CT) Mismatch Rate, which gives the average probability across a set of patients that a mismatch would occur between the patient and donor when a high resolution confirmatory typing is performed on the ambiguously-typed donors in a uniformly-typed registry.

We show that entropy can be used to objectively compare methods of HLA typing to each other in terms of the information they provide, in the context of each individual population. Our results show that intermediate-resolution single-pass sequence-based typing (SBT) reported in genotype list format contains the least ambiguity and, therefore, the most certainty in allele prediction across all populations. We examine the benefit of using haplotype frequencies in entropy calculations versus allele frequencies. Neighboring HLA and non-HLA genes are highly correlated and major efforts have been directed at describing linkage disequilibrium (LD) across the region [22]–[24]. When certain alleles occur together generally due to linkage disequilibrium between them, some ambiguity can be inferred away using this linkage information, which is inherently contained in haplotype frequencies. Our results show that using population haplotype frequencies immensely reduces the ambiguity present in HLA typing. This demonstration allows HLA typing methods to be objectively evaluated in the practical context of a matching algorithm that uses haplotype frequencies to predict probabilities of allele level matches between a patient and list of potentially matched donors. It is hoped that this analysis can lead to data-driven HLA typing resolution strategies for registry donor and cord-blood unit (CBU) typing.

## Materials and Methods

### Typing Formats

The naming of HLA alleles is standardized and regulated by the World Health Organization (WHO) Nomenclature Committee for Factors of the HLA System [25]. Each allele name starts with the locus name (e.g. A, B, DRB1, etc.) followed by at least two sets of digits; the first set corresponds to the allele family, often associated with serologically-defined antigen groups, and the second set corresponds to a specific protein within the group (e.g. allele A*02:01 is found on locus A, belongs to the “02” allele family and encodes a protein named “01”). If necessary, longer allele names are assigned, up to a total of four sets of digits; the third set of digits is used to show synonymous substitution within the coding region, and the fourth set of digits is used to denote differences in non-coding regions of the gene.

Before DNA-based HLA typing methods were developed, serological testing identified sets of alleles with similar reactivity. Two-digit level resolution is the lowest HLA typing resolution reported by typing laboratories today. For these lower-resolution formats, alleles in the same family as A*01:01 are reported as A1 using serological methods (abbreviated SERO), or as a truncated result of intermediate-level DNA-based typing (referred to as DNA2 in this text) as A*01 or A*01:XX.

A commonly used intermediate-resolution format is the one using NMDP *allele codes* [26].

Sequence-specific oligonucleotides (SSO) typing results are reported in this format for this study, where each allele code represents two or more alleles. For example, an allele reported as A*01:AB can be either A*01:01 or A*01:02, and an allele reported as A*26:JGSJ can be any of the following three: A*25:13, A*26:01, A*26:52. Therefore, the number of combination for an ambiguous allele pair reported in this format increases multiplicatively, that is, the allele pair (A*01:AB, A*26:JGSJ) will have six possible pairwise combinations (A*01:01, A*25:13 or A*01:01, A*26:01 or A*01:01, A*26:52 or A*01:02, A*25:13 or A*01:02, A*26:01 or A*01:02, A*26:52). Ambiguous sequence based typing (SBT) is reported in the format of genotype lists for this study, that is, in the form of several possibilities for pairs of alleles an individual carries (A*24:02,A*68:01 or A*24:03,A*68:01 or A*24:04,A*68:01). Because in single-pass SBT results, some ambiguous genotype lists cross several allele families, allele codes could be used to represent all typing results. However, genotype list representation has the advantage of showing that some genotype possibilities, added implicitly when compressing to allele code format, are not possible.

### Data Sets

#### Haplotype Frequency Data

We used high-resolution haplotype frequencies generated from unrelated donors from the National Marrow Donor Program (NMDP) database for four principal population categories defined by the United States census: African American (AFA), Caucasian (CAU), Hispanic (HIS) and Asian/Pacific Islander (API) [27]. These categories are referred to as self-described ethnic groups (SIRE), as they are selected by individuals from the NMDP race/ethnicity questionnaire at donor registration. NMDP develops and maintains a repository of several million HLA-typed donors to facilitate hematopoietic stem cell transplantations among unrelated individuals. Table 2 shows populations used and the number of haplotypes, HLA-A, -B, and –DRB1 alleles within each population.

**Table 2:** HLA haplotype frequency data used in this study.

Population	Description	# of 3-locus Haplotypes	# of HLA-A Alleles	# of HLA-B Alleles	# of HLA-DRB1 Alleles
**AFA**	African American	3,049	68	107	59
**CAU**	Caucasian	5,214	97	158	70
**API**	Asian-Pacific Islander	2,157	56	102	62
**HIS**	Hispanic	3,102	75	138	62

This table shows four population groups and their corresponding haplotype frequencies used for the simulation of samples in this study. The data contains frequencies for three-locus haplotypes (A∼B∼DRB1). The table also shows the number of unique HLA-A, HLA-B and HLA-DRB1 alleles present in the haplotypes for each population group.

#### Simulated Typing Results

To generate simulated typings for different HLA typing methods, we first sampled 2 haplotypes from high resolution population haplotype frequency data set [27]. These sampled haplotypes were then “rolled up” from the high resolution typing to a lower resolution typing to emulate how the typing would have appeared using various typing methods. For example, a high resolution haplotype pair with the format:
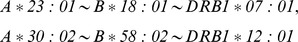
would be rolled up into lower resolution typing (in this case serology) as follows:
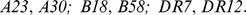
Note that the simulated haplotypes contain neither phase nor allele ambiguity, while the lower resolution typing contains both. Simulated typings of 1,000 individuals were generated for the four broad population groups (AFA, API, CAU, HIS) and four different typing methods (SERO, DNA2, SSO, SBT).

While we use HLA nomenclature Version 3 style formatting to describe HLA alleles in this paper, we simulated the four typing methods for Version 2.28 of the IMGT-HLA database, to more closely match the time in which the typing results used to generate the haplotype frequencies were reported. To generate serologic typing (SERO), we used the HLA dictionary, which allows each HLA allele to be mapped to a serologic equivalent (e.g. B*15:02 maps to B75) [28]. To map alleles to DNA 2-digit (DNA2), we removed all fields from the HLA typing but the first field describing the allele family (e.g. B*15:02 becomes B*15:XX). To simulate SSO typing, given detailed information on the probes present in each SSO kit and the IMGT/HLA database of allele sequences, probe hit tables were computed for all possible combinations of described alleles. Each pair of alleles was mapped to the set of allele pairs that had identical probe hit patterns, then the typing was compressed to NMDP allele code format. For HLA-A and HLA-B loci we used kits described at the 12th International Histocompatibility Workshop [29], and for HLA-DRB1 locus we used a kit described at the 11th International Histocompatibility Workshop [15], [30]. SBT simulation mapped allele pairs to a list of ambiguous genotypes with identical heterozygous sequence in exons 2 and 3 published by IMGT-HLA (http://www.ebi.ac.uk/imgt/hla/ambig.html) and reported the typing in the genotype list format.

### Shannon's Entropy

Shannon's entropy quantifies the amount of uncertainty or disorder associated with a particular system, and is widely used in a variety of applications, such as genetics [31]–[33], data mining [34], molecular biology [35], and imaging [36]. In information theory, entropy is used to measure uncertainty associated with a random variable. Here we used entropy to measure and compare ambiguity associated with the results of various HLA typing methods. Given an ambiguous genotype *X*, Shannon's entropy (*H*) is defined as:
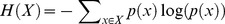
(1)Where 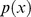 is the relative frequency of a single-locus or multi-locus high resolution genotype 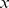.

Entropy can be thought of as the ambiguity or impurity present in a system of interest. If, in a set of typing results for one individual, all of them are equally likely (frequent) then the entropy is the highest as we have the least information to choose the most likely real genotype.

To illustrate the usefulness of entropy in measuring typing ambiguity we show an example of two typing results with the same number of ambiguities (Table 3). Both of these ambiguous typings have six possible pairs of alleles, however, one of them is more pure with respect to the frequency distribution of these combinations, and therefore has a lower entropy (0.014 versus 1.676, with a mean entropy of 0.54 for ambiguous typing results in this sample). Note that entropy depends on the size of the set as well as the distribution of frequencies in the set. If *H_n_* is the entropy of *n* typings, then it is maximal for outcomes of equal frequencies, that is, 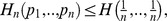 and it increases with the number of equally frequent typings, that is, 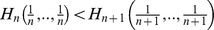. Therefore, depending on the distribution of frequencies and the number of possible alleles for an ambiguous allele pair, typing results can have drastically different entropies.

**Table 3:** An illustration of two ambiguous typing results with the same number of possible allele sub-types and different level of ambiguity as measured by entropy.

Typing 1			Typing 2		
Ambiguities	Relative Frequency	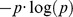	Ambiguities	Relative Frequency	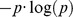
B*5702/B*5801	0.0923	0.3172	DRB1*0301/DRB1*1301	0.9989	0.0016
B*5702/B*5802	0.0832	0.2985	DRB1*0301/DRB1*1327	0.0005	0.0056
B*5702/B*5804	0.0001	0.0016	DRB1*0304/DRB1*1301	0.0002	0.0024
B*5703/B*5801	0.4331	0.5229	DRB1*0304/DRB1*1327	0.0000	0.0000
B*5703/B*5802	0.3907	0.5297	DRB1*0306/DRB1*1301	0.0004	0.0044
B*5703/B*5804	0.0006	0.0063	DRB1*0306/DRB1*1327	0.0000	0.0000
**H = 1.676**			**H = 0.014**		

Shown here are typing results for two simulated subjects. They each have six ambiguous sub-types (allele-pairs), but very different entropies. Typing 1 has entropy H = 1.676 and Typing 2 has entropy H = 0.014. Both of these typings come from the same population sample (African American) in which the mean allele entropy for this simulated sample is H = 0.54.

### Entropy Calculations using HLA Frequencies

The HLA haplotype frequencies we used in this study were estimated by the expectation-maximization algorithm (EM) described in [37]. To obtain the allele frequency of allele 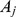 from haplotype frequencies we simply summed the frequencies over all haplotypes in the given population group that included that allele, that is:
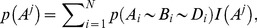
(2)where *A, B*, and *D* are typed loci, *N* is the total number of haplotypes and 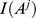 is an indicator function that takes value of 1 (0) when a haplotype contains (does not contain) allele 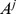. For each ambiguously typed locus, we generated all possible pairs of alleles 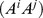, and computed their respective frequencies as follows:
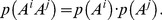
(3)In the first set of experiments, we used these allele pair frequencies derived from allele frequencies in entropy calculations to compute ***allele entropy*** for each locus.

To compute locus entropy using haplotype frequencies, or ***haplotype entropy***, we do the following. For each ambiguously typed three-locus genotype, *g = (AaBbDd)*, we generated all possible haplotype pairs and their frequencies using imputation as described in [37]. To compute the entropy of a particular locus, we obtained frequencies for each unique allele pair on that locus, say 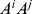, by summing over all frequencies of haplotype pairs generated for the given genotype, that contain the given allele pair, that is
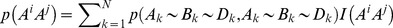
(4)where N is the number of generated haplotypes and 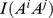 is an indicator function that takes value of 1 (0) when a haplotype pair contains (does not contain) allele pair 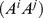. We then used these allele pair frequencies derived from haplotype frequencies to compute *haplotype entropy* in the same manner as described for the case of *allele entropy*.

As a side note, an ambiguous typing with many possible alleles at each locus can result in a large combinatorial number of possible haplotype pairs. Given a fully heterozygous case of three-locus un-phased genotype with 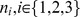 possible alleles at each locus, the number of possible haplotypes is equal to 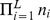 and the number of phased haplotype pairs is equal to 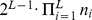, where L is the number of loci, in this case 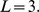 In these equations we assume the heterozygosity of all loci, since the estimated numbers would be smaller for a homozygous case in which some HLA loci have the same alleles on both chromosomes. For typings including five or six HLA loci and high allelic ambiguity, the number of phased haplotype pairs can grow into billions.

### Confirmatory Typing Mismatch Rate

Besides objective evaluation of typing methodologies employed in typing the HLA region, using the entropy approach to measure ambiguity has another application from a clinical perspective, namely its direct relationship to confirmatory typing (CT) mismatch rates. For a given patient, high resolution CT is done to confirm the patient-donor match from a selected set of donors. A case where the high resolution typings mismatch is called a CT mismatch. We compute CT mismatch rates on the same simulated donor sample by comparing the ambiguous typing and the exact haplotype pair that was used to generate that ambiguous typing. As described in a previous section, each ambiguous genotype can generate multiple HLA haplotype pairs, the true one being the haplotype pair we used to simulate the ambiguous typing. The CT mismatch rate for each locus is computed as the summation of frequencies of all allele pairs (computed using Equation (4)) that do not match the corresponding allele pair of the haplotypes used to simulate the donor typing. This is the probability that a selected donor will not be the exact match for the given patient.

## Results

**Figure 1:**
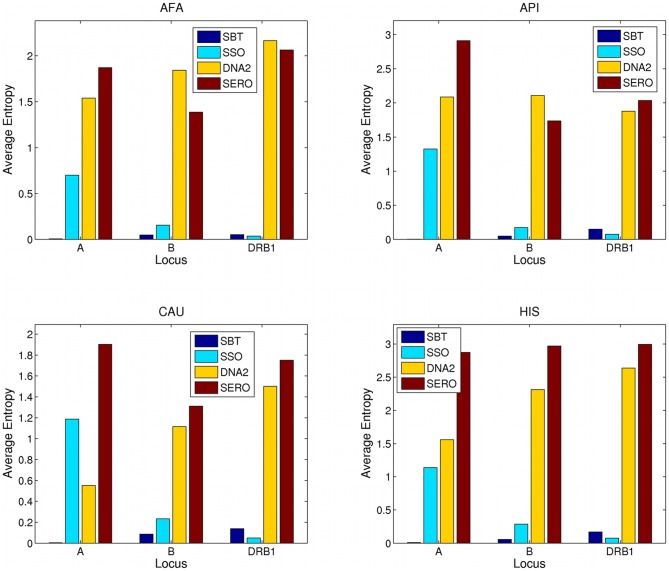
Average allele entropy. This figure shows locus entropies obtained for SBT, SSO, DNA2 and SERO typing formats, within each population and for three HLA loci using allele frequencies, that is, the *allele entropy*. The four panels correspond to four populations: AFA (African American), API (Asian-Pacific Islander), CAU (Caucasian) and HIS (Hispanic), respectively, from left to right, top to bottom. The y-axis shows entropy averaged across 1000 simulated donor typings, and the x-axis corresponds to the HLA locus that the entropy is measured for (HLA-A, HLA-B and HLA-DRB1). The color represents the typing methods used for typing: SBT (single pass sequence-based typing), SSO (sequence-specific oligonucleotides), DNA2 (two-digit allele family level DNA-based typing) and SERO (serological typing).

Locus entropies obtained for SBT, SSO, allele family level DNA2 and SERO typing methods are shown in Table 4, averaged over the three loci (HLA-A, -B, -DRB1) and within each population using allele frequencies (*allele entropy*). SBT had the lowest entropy and therefore the least inherent ambiguity when it comes to resolving HLA alleles at the locus level, across all populations. As expected, serology produced the lowest resolution typing and had the highest entropy. SSO typing was far more ambiguous than SBT across all evaluated datasets, reflecting both SBT's more complete coverage of polymorphic positions in the exons and the benefits of using genotype list format for SBT typings rather than the NMDP allele code format we used for SSO. Figure 1 shows the average allele entropy for each locus for AFA, CAU, HIS and API population groups. The ranking of the typing methods with respect to the least amount of ambiguity across all populations was: SBT, SSO, allele family level DNA2, SERO.

**Table 4:** Average *allele entropy* for all typing methods and all population groups.

	AFA	API	CAU	HIS
**SBT**	0.0354	0.0668	0.0766	0.0787
**SSO**	0.2974	0.5247	0.49	0.5004
**DNA2**	1.8501	2.023	1.056	2.1709
**SERO**	1.7735	2.2282	1.6548	2.9471

This table shows the locus entropy for SBT, SSO, DNA2 and SERO typing methods for all four populations using allele frequencies and averaged over the three loci, HLA-A, -B, -DRB1.

**Figure 2:**
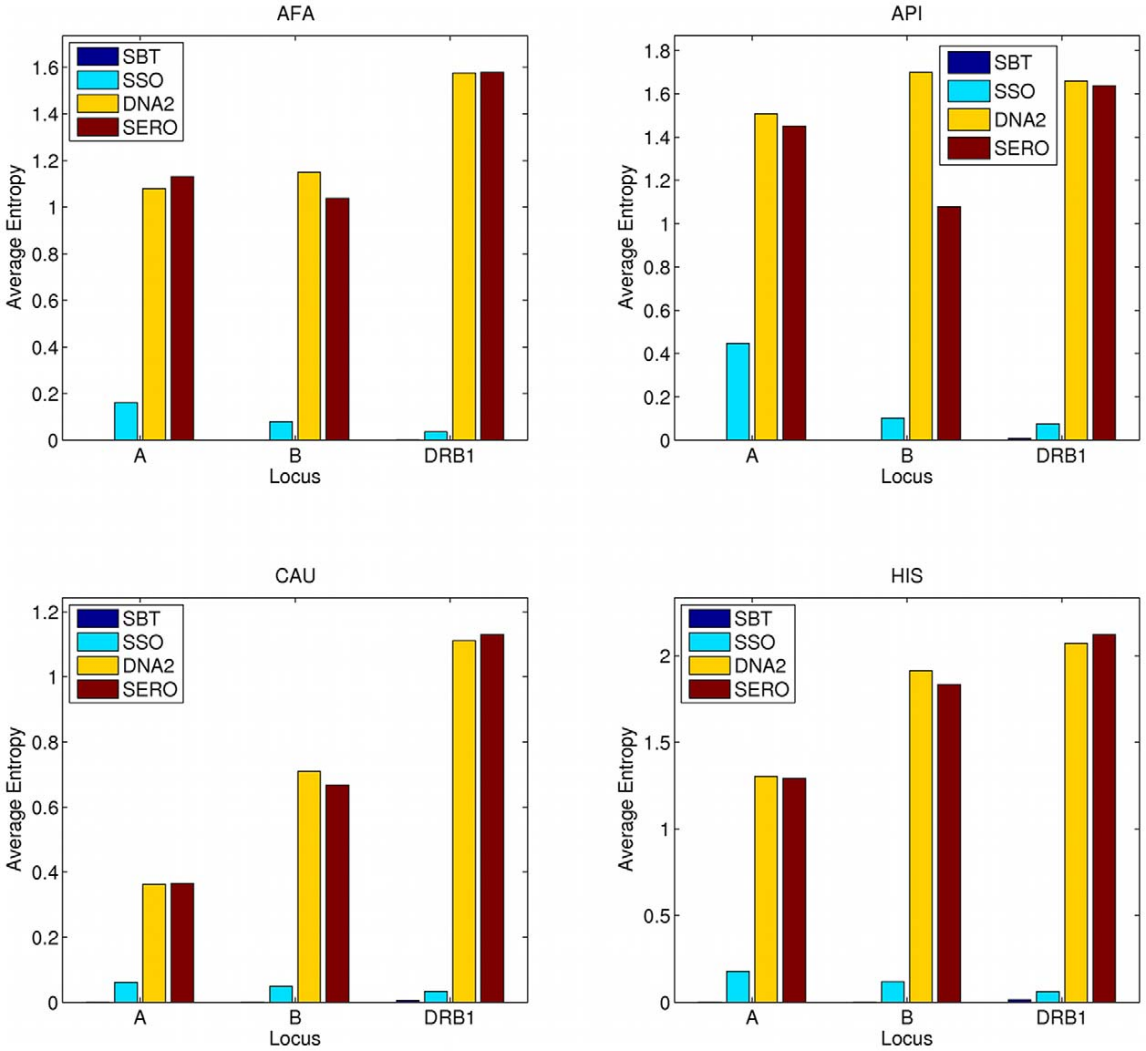
Average haplotype entropy. This figure shows locus entropies obtained for SBT, SSO, DNA2 and SERO typing formats, within each population and for three HLA loci using haplotype frequencies, that is, the *haplotype entropy*. The four panels correspond to four populations: AFA (African American), API (Asian-Pacific Islander), CAU (Caucasian) and HIS (Hispanic), respectively, from left to right, top to bottom. The y-axis shows entropy averaged across 1000 simulated donor typings, and the x-axis corresponds to the HLA locus that the entropy is measured for (HLA-A, HLA-B and HLA-DRB1). The color represents the typing methods used for typing: SBT (single pass sequence-based typing), SSO (sequence-specific oligonucleotides), DNA2 (two-digit allele family level DNA-based typing) and SERO (serological typing).

**Figure 3:**
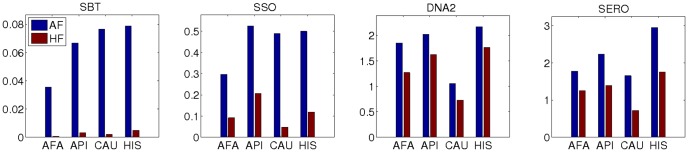
Comparison of average per-locus entropies obtained from allele and haplotype frequencies. This figure shows the comparison between *allele entropy* and *haplotype entropy* computed for four typing methods: SBT (single pass sequence-based typing), SSO (sequence-specific oligonucleotides), DNA2 (two-digit allele family level DNA-based typing) and SERO (serological typing), respectively from left to right. Allele entropy was computed using allele frequencies (AF) and genotype entropy was computed using haplotype frequencies (HF). The y-axis shows the average entropy values, and the x-axis shows the four continental populations for which the entropy was computed: AFA (African American), API (Asian-Pacific Islander), CAU (Caucasian) and HIS (Hispanic), respectively. In all figures, the locus entropy is averaged across the three loci, HLA-A, -B, -DRB1.

When we used haplotype instead of allele frequencies, we got the same ambiguity ranking (Table 5) for average locus entropies obtained for SBT, SSO, and DNA2 typing methods (*haplotype entropy*). However, a dramatic decrease in entropy occurred across all typing methods when we used haplotype instead of allele frequencies. For example, the allele entropy of SSO typing in Caucasian group is 0.49 while the haplotype entropy is an order of magnitude lower at 0.0477. This decrease is due to some ambiguity being resolved by LD information provided in haplotype frequencies, and is successfully captured by Shannon's entropy. Figure 2 shows the average haplotype entropy for each locus and each population separately. The LD information contained in haplotype frequencies reduces the entropy considerably compared to only using allele frequencies, demonstrating that this strategy for identifying matched unrelated donors has a significant positive impact. Figure 3 shows the comparison between allele and haplotype entropies for each typing method and within each population group. This result also demonstrates the utility of imputation algorithms that generate population haplotype frequencies to more accurately predict the likelihood of allele match for stem cell registry matching algorithms.

**Table 5:** Average *haplotype entropy* for all typing methods and all population groups.

	AFA	API	CAU	HIS
**SBT**	5.30E-04	0.0031	0.002	0.005
**SSO**	0.0923	0.2074	0.0477	0.1195
**DNA2**	1.2685	1.6219	0.7277	1.7633
**SERO**	1.2488	1.3889	0.7205	1.7495

This table shows the locus entropy for SBT, SSO, DNA2 and SERO typing methods for all four populations using haplotype frequencies and averaged over the three loci, HLA-A, -B, -DRB1.

To demonstrate the impact of typing method ambiguity in a clinical setting we computed CT mismatch rates, which give the average probability that a mismatch would occur between a patient and donor when high resolution confirmatory typing is performed on the ambiguously typed donors in a uniformly typed registry. CT mismatch rates computed on the same sample of 1000 simulated donors are shown in Table 6. We can see a direct correlation between CT mismatch rates and entropy computed across typing methods dimension (Pearson's correlation coefficient between CT mismatch rates and haplotype entropy is ρ = 0.96, and between CT mismatch rates and allele entropy is ρ = 0.92). For SSO typing we found an average haplotype entropy of 0.05 and a 1.31% CT mismatch rate, while for SBT typing, we found an average haplotype entropy of 0.002 and a 0.08% CT mismatch rate, in the CAU population group. In a hypothetical donor registry with uniformly typed donors, choosing a typing method with smaller entropy across a given population may result in smaller mismatch rates during the confirmatory typing phase and more certainty in a selected set of donors. This CT mismatch rate gives us a more intuitive clinical interpretation of these entropy scores. An important assumption when computing CT mismatch rates is that all donors in the registry are typed with the same method. This is generally not the case, and as donors with better typing accrue, CT mismatch rates are expected to decrease over time.

**Table 6:** *Confirmatory typing (CT) mismatch rates* for all typing methods and all population groups.

	AFA	API	CAU	HIS
**SBT**	2.0135e-04	9.3340e-04	8.0012e-04	0.0015
**SSO**	0.0236	0.0530	0.0131	0.0324
**DNA2**	0.3792	0.4286	0.4069	0.4366
**SERO**	0.3755	0.3724	0.1957	0.4334

This table shows the expected confirmatory typing (CT) mismatch rates for SBT, SSO DNA2 and SERO typing formats and four populations averaged across all three loci (HLA-A, -B, DRB1). CT mismatch rates describe the probability that a mismatch would occur between a patient and donor during high resolution confirmatory typing on the ambiguously typed donors in a uniformly typed registry.

## Discussion

We have shown that entropy can be used to objectively compare methods of HLA typing in terms of the information they provide. The calculation of per-locus entropy using haplotype frequencies has a direct application in measuring the impact of using haplotype frequencies to predict the likelihood of allele match for stem cell registry matching algorithms. The LD information contained in haplotype frequencies reduces the entropy considerably compared to using allele frequencies, showing that this strategy for identifying matched donors has a significant positive impact.

No objective quantitative comparisons between SBT and SSO methods have been available to date. Typing laboratories may choose SSO methods over SBT methods primarily based on cost savings achieved due to easier set-up, staff training, pre-packaged kits, and automation. However, these apparent cost savings may have a price of higher typing ambiguity. We have shown that single-pass SBT typing performs far better at distinguishing alleles compared to mid-1990's-era SSO typing. However, currently available SSO typing kits used for recruitment typing have more oligonucleotide probes and thus are able to distinguish more alleles, which may result in entropy as low as that of single-pass SBT. Given equal cost, registries should utilize laboratories that employ HLA typing methods that achieve lower entropy for their population.

Our objective measure of typing ambiguity can be advantageously applied to the continual improvement of all methods of HLA typing. Design of SSO kits could be done in silico using population haplotype frequencies and sequence information from the IMGT-HLA database [8]. SSO kits are designed to distinguish between the most common alleles in a population. In United States and Europe, populations of European origin predominate, and therefore some alleles common in minority populations may not be distinguished in some kits. Given a fixed number of probes, the SSO kit that provides the lowest entropy in a population could be considered optimal.

As new alleles are discovered, SSO kits are often altered to add more probes so that typing results do not cross allele families and thus meet current guidelines for acceptable recruitment typing. These new probes will not decrease entropy appreciably as the frequency of a newly discovered allele tends to remain very low.

Because of sample size limitations, many of the rare alleles described in IMGT-HLA were not observed in our samples. However, rare alleles do not have a significant impact on entropy calculations. Owing to the logarithmic nature of Shannon's entropy, an allele with a very small frequency *p* contributes 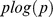 to the resulting entropy. This quantity approaches zero for very small values of *p*. More formally, 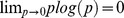. This property of the entropy guarantees that potential underestimation of frequencies of rare alleles not included in the population groups in Table 2 will only slightly underestimate the typing ambiguity. Larger frequency-generating sample sizes available in the future will serve to eliminate this issue.

Our methods of HLA typing method evaluation can also be applied to next-generation sequencing technologies. Recently the Roche 454 sequencing platform has been employed for HLA typing in research rather than recruitment [38], [39]. The 454 platform has relatively longer read lengths that can clonally type entire exons without intra-exonic phase ambiguity. However, intronic regions are not amplified by this platform, thus the system lacks the inability to phase across exons leading to some remaining genotypic ambiguity, which would be reflected in entropy calculations. Meanwhile, other next-generation sequencing platforms more commonly used for whole genome sequencing use a shotgun approach for sequence coverage that includes intronic regions of HLA genes [40]. The Illumina platform uses short reads and high read depth, and there is a potential for HLA typing ambiguity to vary between sequencing runs on the same sample because of differences in read coverage, and thus success with assembly [41]. With the ambiguity of current SBT methods caused by the reading of heterozygous sequence from two chromosomes simultaneously, a future technology that would allow for a single chromosome to be read clonally could eliminate haplotype ambiguity [42]. It is important to note that many genome-wide studies in practice do not make HLA allele calls because the polymorphism of the HLA system requires specialized bioinformatics analysis unique to these genes [43].

A consideration specifically related to the HLA typing method is the representation of the ambiguous allele data derived from the HLA typing, which was also measured using entropy. The 2-digit DNA typing resolution is in practice a result of incomplete reporting of SSO, SSP, or SBT typing data. The higher entropy of this type of data shows the value in reporting the complete information available from the HLA typing platform rather than rounding to the allele family level. The genotype list representation yields a slightly lower entropy than the NMDP allele code representation. Genotype list representation allows for the exclusion of some genotypes that have been ruled out by the HLA typing method, but would still be included in the Cartesian product of the alleles listed in the NMDP allele codes.

Note that, in some populations, the HLA-B locus presents higher values of entropy when typed using 2-digit DNA methods than when typed at the serological level (Figure 1). This is likely due to the fact that some 2-digit DNA allele families contain alleles from multiple serologically defined categories. For example, serological antigens exist to split alleles in the HLA-B15:XX family into B62, B63, B75, B76 and B77, and HLA-B40:XX family into B60 and B61, while 2-digit DNA typing does not distinguish between them.

This analysis provides a path for defining acceptable HLA typing for recruitment as minimum requirements for entropy scores as a measure of typing ambiguity and for HLA data representation guidelines as a way to ensure that genotype lists are reported. Single-pass or highly automated SBT can result in HLA typings that cross allele families, which does not meet current minimum standards for recruitment typing at NMDP, yet we show that it provides a high-quality low-entropy HLA typing. In fact, we had to use the genotype list representation for the simulation of single-pass SBT typings because some allele combinations result in HLA typings for which no NMDP allele codes have been created due to required minimum standards that HLA allele codes do not generally cross allele families [44]. Requiring laboratories to resolve ambiguous alleles in SBT to meet current NMDP requirements can significantly increase cost, but does not significantly lower entropy. Developing a standard for laboratory reports of typings that cross allele families would thus enable a reduction in cost of recruitment typing without a reduction in quality.

We observe variation in entropy for the same HLA typing method between populations and loci. For example, Figure 3 shows lower average entropy of SSO typing results in the CAU sample than in the HIS sample. Some variation is attributed to differences in frequency-generating sample size for a particular population group (in this example simulated Caucasian typings are generated from a larger pool of haplotype frequencies than Hispanic typings, due to a larger availability of Caucasian donors in the registry). The remaining entropy differences can be attributed to the nature and magnitude of HLA genetic diversity in the same group (one can expect HIS population group to be more broadly defined and hence more genetically diverse). The resulting entropy can also depend on LD patterns, the location and number of DNA polymorphisms, and the shape of the allele frequency distribution in the populations.

In addition to absolute differences in entropy between populations, we also observed differences between population groups in the effectiveness of using haplotype frequencies in decreasing haplotype entropy. Having higher levels of LD can improve the predictive capability of haplotype frequencies, and so African population samples with lower LD could have higher entropy than European populations, with higher LD, for this reason. In the opposite direction, higher HLA diversity would lead to higher entropy in African population samples than in European samples. The API sample may have relatively higher entropy than other population samples because the API frequency distribution constitutes an average of the frequency distributions of multiple distinct populations, and thus may be skewed more towards rare types than other populations in this study. If API entropy were evaluated using more detailed race subcategories (e.g. Japanese, Korean, Filipino, etc.), we would expect lower entropy values because the HLA diversity of each respective sub-region would be lower. The size of the population sample used to generate haplotype frequencies also plays a role in the entropy calculations in that a relatively larger sample, as we had for CAU compared to the other races, would give higher entropy. Because of these multiple confounding factors affecting entropy, we urge caution in using entropy as a measure to compare the HLA characteristics between samples of different ancestry. There are some caveats in that the simulation framework implicitly has no sampling error or estimation error in the haplotype frequencies. In practice, uncertainty in the frequency estimates will lead to higher entropy, so our results should be treated as a practical lower bound.

For interpretation of between-locus entropy differences, we turn to the history of HLA nomenclature in that the allele families and serologic types were defined primarily using European samples. The naming of allele families was based loosely on serologic categories, and at some point in history newly discovered serologic patterns were no longer used to split up allele families. The discovery of new alleles also has an impact on entropy in that some populations have not been well-characterized for HLA and some individuals may have as yet un-described alleles that can result in some hidden entropy. In evaluating entropy at the locus level, we see that at the allele family level, the HLA-DRB1 locus has a higher entropy than HLA-A and HLA-B loci. The number of allele families defined for HLA-A and HLA-B is higher than that of HLA-DRB1, giving a lower entropy for typing resolution at the 2-digit or serologic levels, all else being equal.

Stem cell registries have been accruing HLA typing results for over 25 years, with continual advancement in typing methods during this period. The proportion of donors typed by each method changes over time in a searchable registry due to new donor recruitment, roll-off of donors exceeding the maximum age, reporting of primary HLA data, prospective typing, and high resolution typing on behalf of patients. With analysis of changes in HLA typing data for each donor over their time on the registry, it becomes possible to chart decreasing entropy in HLA typing over time and determine which typing methods were primarily responsible for this decrease. Entropy could also be applied as a selection factor for prospective typing projects in which some donors are upgraded to lower ambiguity typings.

In summary, the application of Shannon's entropy as a measure of HLA typing ambiguity has benefits throughout the lifecycle of HLA typing: in reagent design, lab reporting standards, donor recruitment typing guidelines, and registry matching algorithm performance evaluation.

## Supporting Information

Text S1 **Frequency Inferred Typing (FIT) Index.** FIT index is proposed as the first attempt to characterization of HLA typing kits. It describes the probability of correct allele pair assignment for an ambiguous typing result, and is calculated as the negative log of the probability of a wrong allele pair prediction.
(DOC)
