# Supplementary material for: Measuring Ambiguity in HLA Typing Methods
Source: PLoS One. 2012 Aug 29;7(8):e43585. doi: 10.1371/journal.pone.0043585 (PMC3430707; doi:10.1371/journal.pone.0043585)
Supplement: Text S1 — Frequency Inferred Typing (FIT) Index. FIT index is proposed as the first attempt to characterization of HLA typing kits. It describes the probability of correct allele pair assignment for an ambiguous typing result, and is calculated as the negative log of the probability of a wrong allele pair prediction. (DOC) [file pone.0043585.s001.doc]

**Supporting Information - Text S1**

**FIT Index**

Let N be the number of allele pairs that share the same typing pattern, and $p_{k},k=1..N$ their relative frequencies obtained by multiplying individual frequencies of the two alleles, that is$p_{k}=p_{k1}\cdot p_{k2}$.

If the frequency of selected allele pair is $p_{j}$then FIT index of the typing outcome is calculated as:

$$FIT=-\log(\sum_{\begin{matrix} k=1 \\ k\neq j \end{matrix}}^{N} p_{k})$$

FIT score is computed on a set of frequencies of all possible allele pairs resulting from ambiguous typing.

Given two sets of ambiguous allele pairs with same frequency of most likely allele pair and largely different sizes and distribution, FIT index will be the same, while entropy will change to reflect different distributions between the two sets of allele pairs. For example, given the following two sets of allele pair frequencies F1=(0.5, 0.1, 0.1, 0.1, 0.1, 0.1) and F2=(0.5, 0.25, 0.25), FIT index will be the same (0.301), and the entropy will be different as to reflect larger ambiguity in F1 (2.16 and 1.5, respectively).
